# Molecular mechanisms and network interactions in radiation-induced lung injury

**DOI:** 10.3389/fphys.2026.1766537

**Published:** 2026-05-18

**Authors:** Jing Liu, Xinying Ma, Hengjiao Wang, Ying Yan, Ying Xu

**Affiliations:** 1 Department of Radiation Oncology, General Hospital of Northern Theater Command, Shenyang, Liaoning, China; 2 Graduate School of Dalian Medical University, Dalian, Liaoning, China

**Keywords:** radiation-induced lung injury, DNA damage, oxidative stress, cellular senescence, molecular mechanisms

## Abstract

Radiation-induced lung injury (RILI) represents a major dose-limiting complication of thoracic radiotherapy, profoundly affecting therapeutic outcomes and patient quality of life. Although RILI is clinically significant, its molecular underpinnings are not fully elucidated, hampering the development of targeted interventions. This review summarizes the current understanding of the molecular mechanisms underlying RILI, with a focus on radiation-induced DNA damage, oxidative stress, regulated cell death, inflammatory and fibrotic signaling, cellular senescence, and immune microenvironment dysregulation. Particular attention is given to the crosstalk among these processes and their roles in driving maladaptive lung repair. We also discuss recent advances in single-cell and spatial omics that have improved the understanding of cellular heterogeneity and spatiotemporal dynamics in RILI, as well as emerging targeted therapeutic strategies. Synthesizing contemporary molecular and cellular insights, this review constructs an integrative framework for deciphering RILI mechanisms and highlights promising avenues for future research and clinical application.

## 1 Introduction

Thoracic radiotherapy is a cornerstone in the management of thoracic malignancies. Despite its efficacy in eradicating tumor cells, collateral damage to surrounding normal tissues remains unavoidable. The lung is particularly radiosensitive and frequently develops treatment-related toxicity following thoracic irradiation, commonly referred to as radiation-induced lung injury (RILI) (Guo et al., 2024). RILI is defined as a serious pathological response in normal lung parenchyma following radiation, characterized by interstitial alterations and functional decline (Wang et al., 2020). This frequent radiotherapy complication markedly compromises treatment success and patient well-being (Hanania et al., 2019). About 10% to 20% of patients exhibit varying degrees of RILI symptoms (Kuipers et al., 2024a). For those treated with IMRT, the incidence of acute grade ≥2 RILI reaches 14.2% (Meng et al., 2019). Approximately 30% of patients develop RP within 2–3 months after radiotherapy (Keffer et al., 2020).

RILI encompasses a spectrum of pathological processes, including radiation pneumonitis (RP) and radiation-induced pulmonary fibrosis (RIPF) (Cravereau et al., 2025). RP usually presents within weeks to months after irradiation and is characterized primarily by acute inflammatory injury (Guo et al., 2024), often accompanied by nonspecific symptoms such as cough, fever, and chest discomfort. Histopathologically, RP is associated with type I alveolar epithelial cell injury, compensatory proliferation of type II alveolar epithelial cells, inflammatory cell infiltration, and disruption of alveolar homeostasis (Yang et al., 2022; Zhang et al., 2025). By contrast, RIPF generally develops months to years after irradiation and reflects aberrant tissue repair, with persistent fibroblast activation, myofibroblast accumulation, extracellular matrix deposition, and progressive architectural remodeling of the lung parenchyma (Yu et al., 2023; Fijardo et al., 2024). These changes may ultimately result in extensive alveolar fibrosis, thereby impairing normal lung function. Importantly, while these conditions are interrelated, accumulating evidence indicates that RP and RIPF are not strictly sequential stages of a single disease continuum (Yu et al., 2023; Cravereau et al., 2025).

The pathogenesis of RILI is highly complex and involves dynamic interactions among injured epithelial and endothelial cells, stromal cells, immune populations, and multiple cytokine networks (Yang et al., 2022; Curras-Alonso et al., 2023). Increasing evidence suggests that RILI is driven by overlapping and mutually reinforcing molecular programs, including radiation-induced DNA damage, oxidative stress, danger-associated molecular pattern (DAMP) release, inflammatory signaling, cellular senescence, and dysregulated repair responses (Yin et al., 2019; Zhang et al., 2021; Yang et al., 2022). In general, acute inflammatory injury is more closely associated with DNA damage, reactive oxygen species (ROS) generation, innate immune activation, and cytokine release (Dasgupta et al., 2023; Guo et al., 2024), whereas chronic fibrotic remodeling is more strongly linked to persistent fibroblast activation, epithelial plasticity, cellular senescence, and sustained transforming growth factor-β (TGF-β) signaling (Yu et al., 2023; Fijardo et al., 2024; Zhu et al., 2024). Distinguishing these temporally and mechanistically dominant processes is essential for understanding disease heterogeneity and for developing phase-appropriate therapeutic strategies. Therefore, rather than a simple linear progression from pneumonitis to fibrosis, RILI is better understood as a network-driven process with temporally overlapping yet mechanistically distinct pathological programs. Given the lack of effective targeted therapies in current clinical practice, a deeper understanding of the molecular basis of RILI is of substantial importance. In this review, we summarize the current molecular mechanisms of RILI, distinguish acute inflammatory from chronic fibrotic drivers, and discuss how their network interactions may guide future therapeutic strategies.

## 2 DNA damage and repair imbalance

### 2.1 Radiation-induced DNA damage initiates inflammatory and profibrotic signaling

Radiation induces tissue damage through direct and indirect mechanisms. Direct ionizing radiation causes DNA lesions, including base loss, SSBs, and DSBs (Giuranno et al., 2019). Indirectly, irradiation promotes the generation of ROS, arising both from water radiolysis and from mitochondrial dysfunction, thereby amplifying oxidative stress and genomic instability (Sotomayor et al., 2024; Whitcomb et al., 2024; Rong et al., 2025). During the acute phase after irradiation, rapid ROS accumulation and lipid peroxidation products cause widespread damage to proteins, nucleic acids, organelles, and extracellular matrix components, leading to extensive injury in alveolar epithelial cells and vascular endothelial cells (Najafi et al., 2018; Liermann-Wooldrik et al., 2025). Persistent or incompletely repaired DNA lesions function as signaling hubs that reshape the injured lung microenvironment. Through sustained DNA damage response (DDR) signaling, mitochondrial stress, and the release of DAMPs, injured cells activate downstream inflammatory and profibrotic pathways, including NF-κB-dependent cytokine production, inflammasome activation, and TGF-β signaling. As a result, resident and infiltrating immune cells such as macrophages, lymphocytes, and neutrophils are recruited and activated, leading to the release of TNF-α, IL-1β, IL-6, and TGF-β1 (Yarosz and Chang, 2018). In parallel, persistent DNA damage may induce cellular senescence in epithelial and endothelial cells, thereby establishing a senescence-associated secretory phenotype (SASP) that further amplifies inflammatory signaling and fibroblast-activating cues (Qian et al., 2023). Experimental studies support this model. Radiation-exposed mice exhibit increased DNA double-strand breaks in the lung, and the persistence of this damage correlates with the progression of pulmonary fibrosis (Beach et al., 2018). Additionally, radiation-induced release of cytosolic DNA fragments may activate the cGAS-STING pathway, thereby amplifying innate immune signaling and contributing to subsequent inflammatory injury and profibrotic remodeling in RILI (Zhao et al., 2025). Thus, DNA damage is not only an initiating insult, but also a sustained upstream driver that, under persistent or maladaptive repair conditions, may contribute to both acute tissue injury and chronic fibrotic remodeling by promoting a self-reinforcing pro-inflammatory and profibrotic niche.

### 2.2 Lung cell-specific repair defect

The alveolar epithelium, which consists of gas-exchanging type I alveolar epithelial cells (AECI) and progenitor type II alveolar epithelial cells (AECII), is highly vulnerable to radiation injury (Aspal and Zemans, 2020; Han et al., 2026). AECII are crucial for repair, serving as precursors for AECI and secreting surfactant to maintain alveolar integrity. Radiation rapidly triggers AECI apoptosis, stimulating a compensatory yet dysfunctional proliferative response from AECII. This maladaptive AECII expansion paradoxically suppresses surfactant production, elevating surface tension and predisposing to pulmonary edema and collapse. Concurrently, radiation induces endothelial dysfunction, catalyzing the overproduction of vasoactive mediators (e.g., leukotrienes, prostaglandins), which enhance vascular dilation and permeability, facilitating inflammatory cell infiltration (Huang et al., 2017). Impaired lung repair after irradiation is not solely a consequence of epithelial cell dysfunction but arises from dysregulated multicellular interactions within the alveolar microenvironment. Injured AECII cells release alarmins and chemokines that recruit and activate immune cells, particularly macrophages (Song et al., 2024). Recruited macrophages, in turn, secrete IL-1β, IL-6, and TGF-β, which not only amplify inflammatory signaling but also inhibit epithelial differentiation and promote fibroblast activation (Guo et al., 2024). At the same time, endothelial barrier disruption enhances leukocyte infiltration and sustains sterile inflammation, further reinforcing epithelial injury and stromal activation. Activated fibroblasts respond to these combined epithelial-immune signals by differentiating into myofibroblasts and producing extracellular matrix components, while also releasing TGF-β1 and other niche factors that reciprocally modulate epithelial cell fate and promote maladaptive epithelial plasticity (Brussow et al., 2023; Cohen et al., 2023). Therefore, radiation-induced failure of lung repair should be understood as a microenvironmental network disorder rather than an isolated defect in a single cell type.

## 3 Oxidative stress and the cell death program

### 3.1 Mechanisms and consequences of ROS bursts

Radiation exposure triggers a rapid and sustained increase in ROS/RNS in epithelial, endothelial, and immune cells, establishing a state of oxidative stress. These reactive species directly damage cellular macromolecules, including DNA, lipids, and proteins, leading to base modifications, strand breaks, and structural instability (Soloviev and Sydorenko, 2024). Radiolysis of water is a primary source of ROS immediately after irradiation, generating superoxide (O_2_^-^), hydrogen peroxide (H_2_O_2_), and hydroxyl radicals (·OH) (Wang et al., 2020). The initial ROS burst is not transient. Radiation-induced mitochondrial dysfunction, particularly electron leakage from the electron transport chain, sustains ROS production and establishes a feedforward amplification loop of oxidative stress (Farhood et al., 2020). This persistent redox imbalance contributes to long-term tissue injury. The deleterious effects of ROS are mediated through the oxidation of critical cellular macromolecules—lipids, DNA, and proteins—which disrupts metabolic harmony and cellular function, steering cells toward senescence or death (Su et al., 2022). At the tissue level, persistent oxidative insult compromises vascular endothelial integrity, dismantles cell-cell junctions, and facilitates the extravasation of inflammatory cells, thereby intensifying the overall inflammatory cascade (Wei et al., 2019). Antioxidant components such as superoxide dismutase (SOD) and glutathione (GSH) can suppress oxidative stress, thereby protecting lung tissue from radiation damage (Raeispour et al., 2022; Tan et al., 2022). Anisodamine (654-2) has been shown to increase SOD and GSH levels in irradiated lung tissue, inhibit oxidative stress, and alleviate RILI (Guo et al., 2023).

Beyond direct cytotoxicity, ROS act as central signaling mediators. Oxidative stress activates multiple redox-sensitive pathways, including MAPK and NF-κB signaling, thereby enhancing the production of pro-inflammatory cytokines and amplifying the inflammatory cascade. Concurrently, increased metabolic demand and oxygen consumption contribute to local hypoxia, which induces VEGF expression and promotes aberrant endothelial responses (Averill-Bates, 2024; Gülow et al., 2024; Rauf et al., 2024). Recent studies indicate that mitochondrial gene dynamics and localized oxidative stress contribute to the overall progression of RILI (Yin et al., 2019). At the organelle level, mitochondrial DNA (mtDNA) is particularly susceptible to oxidative damage. ROS-induced mtDNA injury exacerbates mitochondrial dysfunction, enhances oxidative metabolism, and promotes the release of mitochondrial danger signals, further amplifying inflammatory and profibrotic signaling (Yin et al., 2019). Moreover, radiation-induced DNA damage and oxidative stress activate multiple downstream inflammatory and profibrotic mediators, including TGF-β, PDGF, and IL-1, thereby promoting fibroblast activation and maladaptive tissue remodeling (Yu et al., 2023; He et al., 2025). Collectively, oxidative stress functions as a central bridge linking radiation-induced DNA damage to mitochondrial dysfunction, inflammatory amplification, cellular senescence, and signaling that activates fibroblasts.

### 3.2 Multimodal cell death pathways

Radiation-induced cell death is a key driver of tissue injury and microenvironmental remodeling in RILI. Apoptosis represents a major early response to DNA damage, particularly double-strand breaks, and contributes to the loss of epithelial and endothelial integrity (Bigdeli et al., 2026; Zhou et al., 2026). Therapeutically, bone marrow-derived mesenchymal stem cells (BM-MSCs) have demonstrated protective effects in RILI, primarily through immunomodulatory, anti-inflammatory, and tissue-reparative mechanisms (Hou et al., 2022). Emerging evidence indicates that multiple regulated cell death pathways coexist and interact in a context-dependent manner. ROS overproduction facilitates TXNIP dissociation from thioredoxin, subsequently activating the NLRP3 inflammasome and instigating pyroptosis (Su et al., 2026). The classical inflammasome is pivotal for pyroptosis, potentially participating in RILI development through its classical pathway (Wu et al., 2019). Evidence for pyroptosis in RILI has been supported by recent mouse studies showing activation of the cGAS–STING–NLRP3 axis, together with increased expression of pyroptosis-related mediators such as cleaved caspase-1, IL-1β, IL-18, and GSDMD in irradiated lung tissues, underscoring the contribution of this lytic inflammatory cell death pathway to disease progression (Zhang et al., 2023).Ferroptosis, an iron-dependent form of regulated cell death driven by lipid peroxidation, has also been implicated in RILI. Experimental models demonstrate reduced expression of glutathione peroxidase 4 (GPX4), accumulation of lipid peroxides, and characteristic mitochondrial shrinkage in irradiated lung tissue, supporting the involvement of ferroptosis in epithelial injury (Li et al., 2019b; Li et al., 2019a). The NRF2–Keap1 axis plays a critical protective role in this context. Upregulation of p62 disrupts Keap1-mediated degradation of NRF2, facilitating its nuclear translocation and enhancing antioxidant defenses, thereby suppressing ferroptosis (Li et al., 2022). These cell death pathways do not operate in isolation. They form an interconnected network influenced by oxidative stress, mitochondrial dysfunction, and inflammatory signaling. These regulated cell death programs reshape the injured lung microenvironment by releasing DAMPs, altering cytokine composition, and modulating immune-cell recruitment and fibroblast behavior, thereby converting epithelial and endothelial injury into tissue-level inflammatory and profibrotic responses.

## 4 Inflammatory and fibrotic signaling networks

### 4.1 Early inflammatory initiation

Radiation exposure induces immunogenic cell damage, leading to the release of DAMPs, such as HMGB1, from injured epithelial and immune cells. These DAMPs interact with pattern recognition receptors, including Toll-like receptors (TLR2 and TLR4), thereby activating the NF-κB signaling pathway (Wang and Zhang, 2020). Following irradiation, HMGB1 released from stressed or dying cells can bind to TLR4 on macrophages, triggering NF-κB activation and nuclear translocation. Activated NF-κB promotes the transcription of pro-inflammatory cytokines, including IL-1β, IL-6, and TNF-α (Mei et al., 2018). This DAMP-TLR-NF-κB axis is a master regulator of the initial sterile inflammatory response, creating a cytokine-rich milieu that recruits and activates immune cells. Although this early inflammatory program is temporally distinct from later fibrotic remodeling, persistent epithelial damage and maladaptive repair may allow these inflammatory signals to create a microenvironment permissive for fibroblast activation and extracellular matrix deposition.

### 4.2 The core driving role of TGF-β

Fibrotic remodeling represents a major late pathological phenotype of RILI, characterized by myofibroblast accumulation, persistent extracellular matrix deposition, and aberrant tissue repair. In the irradiated lung, mediators released from damaged epithelial cells and activated macrophages drive the activation and differentiation of resident fibroblasts into matrix-producing myofibroblasts (Zhang et al., 2025). Following radiation exposure, TGF-β is rapidly activated and may remain persistently elevated, acting as a key mediator involved in both early inflammatory responses and chronic fibrotic remodeling, particularly under persistent injury or maladaptive repair conditions. Through the canonical Smad pathway, TGF-β binding to its receptor complex induces phosphorylation of Smad2 and Smad3, which then form a complex with Smad4 and translocate to the nucleus to regulate the transcription of profibrotic genes, including type I and II collagen (Wu et al., 2020). Furthermore, TGF-β is a key regulator of epithelial–mesenchymal transition (EMT), a process in which epithelial cells lose their characteristics and acquire mesenchymal features. EMT has been implicated in promoting myofibroblast accumulation and extracellular matrix deposition, thereby contributing to radiation-induced pulmonary fibrosis (Chen et al., 2024). TGF-β signaling does not act in isolation. In fibrotic tissues, oxidative stress and tissue injury can promote the local activation of latent, extracellular matrix–bound TGF-β, while inflammatory cues and other injury-associated signals can induce TGF-β expression and reinforce fibroblast activation. Together, these converging inputs help maintain a self-sustaining profibrotic microenvironment that supports persistent matrix remodeling and disease progression (Frangogiannis, 2020; de Oliveira Camargo et al., 2021). Therefore, TGF-β is better viewed as a network-integrating mediator that links oxidative stress, inflammatory signaling, epithelial injury, and senescence-associated pathways to fibroblast activation and matrix deposition. Future efforts might need to focus on targeting specific downstream effectors or the crosstalk between TGF-β and other signaling pathways.

### 4.3 Autophagy dysfunction and stem cell exhaustion

Autophagy is activated in RILI in response to multiple radiation-induced stressors, including oxidative stress, DNA damage, and endoplasmic reticulum stress (Chaurasia et al., 2016). In addition, autophagy has emerged as a regulator in RILI. While basal autophagy may exert cytoprotective effects by maintaining cellular homeostasis, dysregulated autophagy has been shown to contribute to fibrotic progression through mechanisms such as enhanced fibroblast activation and extracellular matrix deposition (Lin et al., 2024; Li et al., 2025). Under mild-to-moderate oxidative stress, it serves a cytoprotective role by clearing damaged proteins and mitigating AECII apoptosis. However, persistent or excessive stress can impair autophagic flux, particularly through lysosomal dysfunction, leading to the accumulation of damaged cellular components. This defective autophagy exacerbates oxidative stress, promotes apoptosis and other forms of regulated cell death, and sustains chronic inflammatory signaling, thereby contributing to fibrotic remodeling (Racanelli et al., 2018). In addition, impaired autophagy has been linked to dysfunction and exhaustion of regenerative cell populations, further compromising tissue repair capacity. This dual role of autophagy highlights its therapeutic complexity: enhancing autophagy may be beneficial in early injury phases, whereas targeting dysregulated or excessive autophagy may be required to limit fibrosis in later stages. These findings position autophagy as a key regulator at the intersection of inflammation, cell death, and fibrosis, and suggest that stage-specific modulation of autophagy may represent a promising therapeutic strategy in RILI. Overall, these pathways do not operate in isolation, viewing RILI as an interconnected regulatory network may better capture its biological heterogeneity and provide a more rational basis for mechanism-guided interventions.

## 5 Cellular senescence and immune microenvironment dysregulation

### 5.1 Cell senescence

Cellular senescence, a state of irreversible cell cycle arrest frequently triggered by DNA damage or oxidative insult, is increasingly recognized as a key driver of RILI (He et al., 2019; Su et al., 2021). In particular, radiation-induced senescence in AECII critically impairs lung regenerative capacity, as these progenitor cells lose their ability to self-renew and differentiate into AECI. In parallel, radiation can induce premature senescence in multiple lung-resident cell types, thereby contributing to sustained tissue dysfunction (Citrin et al., 2013).A central feature of senescent cells is the senescence-associated secretory phenotype (SASP), a complex secretome enriched in pro-inflammatory cytokines, growth factors, and proteases. The SASP plays a dual role in RILI pathogenesis. On one hand, it reinforces local inflammation through sustained secretion of mediators such as IL-1, IL-6, and TNF-α. On the other hand, it remodels the tissue microenvironment by activating fibroblasts and immune cells, thereby promoting fibrotic progression (Chung et al., 2019; Hansel et al., 2020; Zhu et al., 2024). The SASP also drives intercellular propagation of senescence. Through paracrine signaling, senescent cells can induce a senescent phenotype in neighboring epithelial cells, endothelial cells, and fibroblasts, amplifying tissue damage in a feedforward manner. This propagation of senescence further compromises epithelial repair and promotes a shift toward maladaptive tissue remodeling (Chung et al., 2019). Experimental studies have demonstrated that targeting senescence can ameliorate RILI. Interventions that reduce senescence burden or eliminate senescent cells, including senolytic therapies, have been shown to attenuate inflammation and fibrosis in preclinical models (Hansel et al., 2021; Mungunsukh et al., 2021; Zhou et al., 2022). Multiple drugs targeting senescence prevention or selective senescent cell clearance (senescence clearance therapies) have demonstrated efficacy in preclinical models for preventing or reversing RILI, offering promising prospects for clinical translation (He et al., 2019). Given its upstream role in coordinating inflammation, immune dysregulation, and fibrotic remodeling, cellular senescence represents a promising therapeutic target for RILI.

### 5.2 Immune microenvironment dysregulation

#### 5.2.1 Macrophage activation and functional heterogeneity

Macrophages are the primary mediators of chronic inflammatory responses and can be categorized into two distinct functional phenotypes (Li et al., 2026). M1 macrophages exert pro-inflammatory effects by secreting cytokines and producing matrix metalloproteinases (MMPs), thereby promoting extracellular matrix (ECM) degradation (Craig et al., 2015; Zhang et al., 2018). M2 macrophages play a crucial role in the pathogenesis of pre-fibrotic clusters in pulmonary fibrosis (Groves et al., 2016; de Leve et al., 2017; Rahal et al., 2018). As a key component of the myeloid innate immune system, macrophages participate in maintaining homeostasis within lung tissue, regulating inflammation, and repairing damage. Their high degree of plasticity results in significant heterogeneity under pathological conditions (Locati et al., 2020). During RILI, macrophage states are dynamically shaped by the local microenvironment. Initially, high ROS levels activate MAPK/NF-κB pathways, skewing macrophages toward an M1 phenotype. These M1 cells, stimulated by Th1 cytokines like IFN-γ, amplify inflammation via prolific release of IL-1β, IL-6, and TNF-α (Li et al., 2021). As injury evolves, shifting redox tones and Th2-derived cytokines (IL-4, IL-13) foster M2 polarization through enhanced ERK/p38 MAPK signaling (Park et al., 2019). These M2 macrophages subsequently orchestrate fibrotic processes by secreting TGF-β and other pro-fibrotic mediators.

During acute injury, pulmonary macrophages and monocyte-derived macrophages may be aberrantly activated by ROS, DAMPs, and cytokines such as TNF-α and IL-6 (Lee et al., 2021). Once polarized toward an M1-like phenotype, these cells exert pro-inflammatory and cytotoxic effects by releasing chemokines and cytokines that amplify local inflammatory responses. This establishes an inflammatory microenvironment, recruiting immune cells such as neutrophils and monocytes to infiltrate lung tissue, thereby triggering chronic inflammation (Li et al., 2021). Concurrently, IL-1β secreted by interstitial macrophages inhibits the differentiation of air-epithelial cells (AECs), resulting in incomplete lung tissue repair and impaired regeneration (Melms et al., 2021). In the chronic phase, persistent alveolar injury and incomplete repair mediate macrophage mitochondrial dysfunction (Dong et al., 2022). Rather than a simple M1-to-M2 transition, macrophage responses in RILI are better understood as a continuum of functional states with overlapping inflammatory and fibrotic properties. Advances in single-cell transcriptomics are beginning to reveal previously unrecognized macrophage subpopulations, highlighting the need for more precise characterization of their roles in disease progression. This dynamic heterogeneity allows macrophages to act as central intermediaries between epithelial/endothelial injury and stromal remodeling, enabling immune responses to influence not only inflammation intensity but also the trajectory of tissue repair and fibrotic progression.

#### 5.2.2 Imbalance in T-cell responses

Adaptive immune responses also contribute to the dysregulated microenvironment in RILI. The balance between Th1 and Th2 responses plays a critical role in determining disease outcome. Th1-derived IFN-γ exerts anti-fibrotic effects by directly restraining fibroblast proliferation and collagen deposition, while concurrently reinforcing M1 macrophage activation and inhibiting Th2 responses (Amini et al., 2019). Th2-associated cytokines, particularly IL-4 and IL-13, promote fibroblast activation and collagen synthesis, thereby favoring profibrotic remodeling (Gillesberg et al., 2025). A Th2-skewed immune milieu, relative to Th1-associated responses, may contribute to persistent tissue remodeling and increased fibrotic susceptibility in RILI (Zheng et al., 2024; Wang et al., 2025b). Regulatory T cells (Tregs), a specialized CD4^+^ T-cell subset, also contribute to RILI pathogenesis in a context-dependent manner. By modulating the Th1/Th2 balance, restraining Th17-associated inflammation, and reshaping the profibrotic immune microenvironment, Tregs may exert either pro-fibrotic or anti-fibrotic effects depending on the temporal stage of injury (Guo et al., 2020; Zheng et al., 2024; Wang et al., 2025b). The role of other T-cell subsets, particularly Th17 and cytotoxic CD8+ T cells, in RILI warrants further investigation, as these populations may also contribute to inflammatory injury and tissue remodeling.

#### 5.2.3 Cytokine networks and immune crosstalk

The irradiated lung microenvironment is characterized by a highly dynamic cytokine network that integrates signals from epithelial cells, endothelial cells, immune cells, and fibroblasts. Radiation-induced cell damage leads to the release of DAMPs, which initiate sterile inflammation and drive the production of a wide array of cytokines and chemokines, including IL-1β, IL-6, TNF-α, TGF-β, CCL2, CCL5, and CXCL8 (Cytlak et al., 2022; Yamaga et al., 2024). Alveolar macrophages, as highly specialized innate immune cells, have their functions and fibroblast differentiation regulated by multiple cytokines. Pro-inflammatory and profibrotic cytokines such as TNF-α and TGF-β can activate fibroblasts, triggering paracrine and autocrine signaling circuits between fibroblasts, endothelial cells, and macrophages (Jiang et al., 2024). Fibroblast differentiation is regulated by TGF-β, PDGF, SDF-1, IL-4, IL-13, and other factors. Understanding RILI therefore requires a systems-level perspective, in which disease progression is driven by the dynamic interplay among multiple cell types and signaling pathways rather than by isolated molecular events. Identifying key regulatory nodes within this network may provide new opportunities for targeted therapeutic intervention.

## 6 Spatiotemporal omics and cellular heterogeneity in RILI

### 6.1 A cell atlas revealed by single-cell transcriptomics

Single-cell transcriptomic approaches have substantially advanced our understanding of RILI by enabling the construction of high-resolution cellular atlases. In recent years, several studies have systematically mapped the pulmonary cellular landscape in animal models of RILI using single-cell RNA sequencing (scRNA-seq). These atlases reveal pronounced heterogeneity and dynamic transitional states across epithelial, endothelial, immune, and stromal cell populations. For example, a study demonstrated that alveolar type II (AT2) cells undergo progressive depletion following fibrogenic irradiation, whereas this phenomenon is less pronounced at non-fibrogenic doses, suggesting that AT2 cell dysfunction may play a critical role in fibrotic progression (Curras-Alonso et al., 2023). Mechanistically, TMEM131-mediated sTRAIL secretion has been shown to induce AT2 cell senescence via the DR5/mTOR axis, providing a molecular explanation for their irreversible loss in RILI (Han et al., 2026). Importantly, scRNA-seq enables the identification of rare but functionally critical cell subpopulations that are often obscured in bulk analyses. These include pro-fibrotic fibroblast subsets, distinct macrophage polarization states (e.g., Fabp4 low/Spp1 high and M2-like macrophages), proliferative CD8^+^ Mki67^+^ T cells, and smooth muscle cells with enhanced ECM deposition capacity (Ma et al., 2022; Yang et al., 2022; Curras-Alonso et al., 2023; Shi et al., 2024). Furthermore, single-cell data facilitate the reconstruction of intercellular communication networks through ligand–receptor interaction analyses, uncovering complex signaling crosstalk among fibroblasts, endothelial cells, and immune cells. This provides a mechanistic framework for understanding cellular coordination during RILI progression (Curras-Alonso et al., 2023; Wang et al., 2025).

### 6.2 Spatial transcriptomics reveals microenvironmental heterogeneity

Spatial transcriptomics complements single-cell approaches by preserving tissue architecture and spatial context, thereby enabling the mapping of gene expression within intact lung microenvironments. Direct spatial-transcriptomic evidence in RILI remains limited at present, reflecting the early stage of application of this technology in radiation-induced lung disease. Nevertheless, studies in related fibrotic lung disorders, particularly idiopathic pulmonary fibrosis (IPF), have demonstrated the value of spatially resolved profiling for identifying region-specific pathogenic niches and linking molecular programs to histopathological architecture. Jaeger et al. (2022) identified a KRT17-high airway basal cell niche enriched around fibrotic lesions in IPF lungs, these cells accumulate around fibrotic lesions and promote disease progression. In this context, spatial transcriptomics may help define the anatomical boundaries between inflammatory and fibrotic regions, characterize site-specific epithelial, immune, and stromal interactions, and clarify how local tissue contexts shape maladaptive repair after irradiation. By directly linking molecular signatures to histopathological features, this approach may offer important insights into the spatial organization of pathogenic processes in RILI as direct evidence continues to emerge.

### 6.3 Multi-omics integration and temporal dynamics

Integrated multi-omics analyses, particularly when combined with longitudinal sampling, offer a systems-level perspective on the temporal evolution of RILI. A study integrated transcriptomic, proteomic, and metabolomic datasets across 4, 8, and 16 weeks post-irradiation, revealing time-dependent activation of pathways related to xenobiotic metabolism and ECM–receptor interactions, and identifying Ces2e as a potential early prognostic biomarker (Wu et al., 2026). These integrative strategies help distinguish primary pathogenic drivers from secondary responses and uncover key regulatory circuits to sustained tissue injury. Complex computational algorithms such as CellChat and RNA-Rate integrate diverse data types, enabling the identification of key regulatory hubs and potential therapeutic targets. Wang et al (Wang et al., 2025). identified NETosis as the most enriched programmed cell death mode in RILI by integrating bulk and single-cell datasets, and discovered that communication between neutrophils and macrophages is enhanced via signaling axes such as SPP1-CD44, driving M2 macrophage polarization. These mechanistic insights have also informed therapeutic strategies. A bifunctional anti-PD-1/TGF-β antibody has been shown to alleviate RILI by suppressing both TGF-β/Smad signaling and NETosis (Wang et al., 2025a), while FLASH radiotherapy appears to protect pulmonary progenitor cells and limit radiation-induced senescence (Fouillade et al., 2020).Overall, the integration of single-cell transcriptomics, spatial profiling, and longitudinal multi-omics analyses has shifted RILI research from a bulk tissue-level description toward a cell-resolved, mechanism-driven framework. This paradigm shift not only deepens our understanding of cell fate decisions following radiation exposure but also provides a foundation for the development of targeted strategies to preserve critical cell populations and modulate pathogenic cellular interactions.

## 7 Therapeutic targeting and clinical translation

### 7.1 Targeting the TGF-β signaling axis: mechanistic and clinical evidence for pirfenidone

Targeting the TGF-β signaling axis represents a cornerstone strategy in the treatment of RILI, directly linking molecular mechanisms of fibrosis to therapeutic intervention. Pirfenidone (PFD), a well-established antifibrotic agent, exerts its effects primarily through modulation of TGF-β–driven pathways. In preclinical models of RIPF, PFD significantly attenuates fibrosis by reducing M2 macrophage infiltration and suppressing TGF-β1/Smad3 signaling in both alveolar epithelial and vascular endothelial cells. Mechanistically, PFD inhibits IL-4/IL-13-induced M2 polarization *in vitro*, leading to decreased expression of profibrotic mediators such as ARG-1 and TGF-β1, partly via downregulation of NF-κB p50 (Ying et al., 2021). In human lung organoid models, PFD reduces irradiation-induced expression of key fibrotic markers, including TGF-β, α-SMA, and COL1A2, further validating its translational relevance (Park et al., 2025). Clinically, PFD has been shown to slow lung function decline in IPF, providing a strong rationale for its application in RIPF. Beyond TGF-β inhibition, PFD also modulates oxidative stress and inflammatory pathways, suggesting a multi-layered mechanism of action (Babariya et al., 2024). Notably, its regulatory effects extend to other profibrotic signaling networks, including Wnt/β-catenin and Hippo pathways, as demonstrated in post-COVID-19 fibrosis models (Al-Kuraishy et al., 2022). In a randomized trial involving 134 patients with Grade 2–3 RILI, the combination of pirfenidone and corticosteroids resulted in an 8.0% increase in DLCO% from baseline, compared with a 2.4% decrease in the control group (Hou et al., 2025). These findings underscore a critical transition from empirical symptomatic management to mechanism-based precision therapy.

### 7.2 Multi-target tyrosine kinase inhibition: mechanistic and clinical evidence for nintedanib

Nintedanib, a multi-target tyrosine kinase inhibitor acting on VEGFR, PDGFR, and FGFR, represents another key example of mechanism-based therapy in RILI (Pitozzi et al., 2025). Nintedanib suppresses inflammation and fibrogenesis through modulation of the PI3K/Akt/mTOR pathway and inhibition of CCR2^+^ monocyte recruitment, thereby attenuating immune cell–mediated tissue injury (Pan et al., 2023; Farooq et al., 2024). In addition, recent evidence suggests that nintedanib exerts senolytic-like activity via STAT3 inhibition, linking it to emerging concepts of cellular senescence in RILI pathogenesis (Cho et al., 2022). Clinical studies further support its therapeutic value. A Phase II randomized, double-blind, placebo-controlled trial involving patients with Grade ≥2 radiation pneumonitis demonstrated that the 1-year exacerbation-free rate was significantly higher in the nintedanib plus prednisone group compared with the placebo group (Rimner et al., 2023). Additional studies indicate that nintedanib is particularly beneficial in patients who are not suitable for corticosteroid therapy (Itano et al., 2023; Kuipers et al., 2024b). Moreover, novel delivery strategies, such as inhaled formulations, may enhance local drug retention and reduce systemic toxicity (Togami et al., 2022).

### 7.3 Mechanism-oriented combination therapy strategies

Ionizing radiation-induced cellular senescence, particularly in alveolar type II (AT2) cells, is increasingly recognized as a key driver in the initiation and progression of RIPF (Zhou et al., 2022). Accordingly, targeting cellular senescence has emerged as a promising therapeutic strategy. Recent studies have demonstrated improved survival outcomes with senolytic agents such as CSP7, especially when used in combination with established antifibrotic drugs like pirfenidone or nintedanib (Fan et al., 2025). In a mouse model of radiation-induced pulmonary fibrosis, the Bcl-2/xL inhibitor ABT-263 selectively cleared senescent AT2 cells and successfully reversed established pulmonary fibrosis (Pan et al., 2017). Given the multifactorial pathogenesis of RIPF, mechanism-oriented combination therapies are increasingly regarded as a rational and effective strategy to address the limitations of monotherapy. By concurrently targeting multiple pathogenic pathways—including TGF-β signaling, immune dysregulation, and cellular senescence—combination approaches may achieve synergistic therapeutic effects. For example, the combination of pirfenidone with metformin and BM-MSCs has demonstrated superior antifibrotic efficacy compared to single-agent treatments (Abd Elhamid et al., 2026). Similarly, nintedanib combined with vardenafil exhibits synergistic suppression of fibrotic progression (Bourne et al., 2021). Future directions in RILI therapy include the development of advanced drug delivery systems (e.g., inhaled formulations), computational drug repurposing strategies, and the exploration of bioactive natural compounds such as cryptotanshinone (Togami et al., 2022; Li et al., 2023; Farh et al., 2025). The identification of predictive biomarkers will be essential for guiding personalized treatment strategies. The translation of these approaches into clinical practice will require robust and clinically relevant preclinical models to ensure both efficacy and safety (Hsia et al., 2023). Collectively, these advances highlight the potential of mechanism-based combination strategies to improve therapeutic efficacy.

## 8 Summary, controversies and future directions

**Figure 1:**
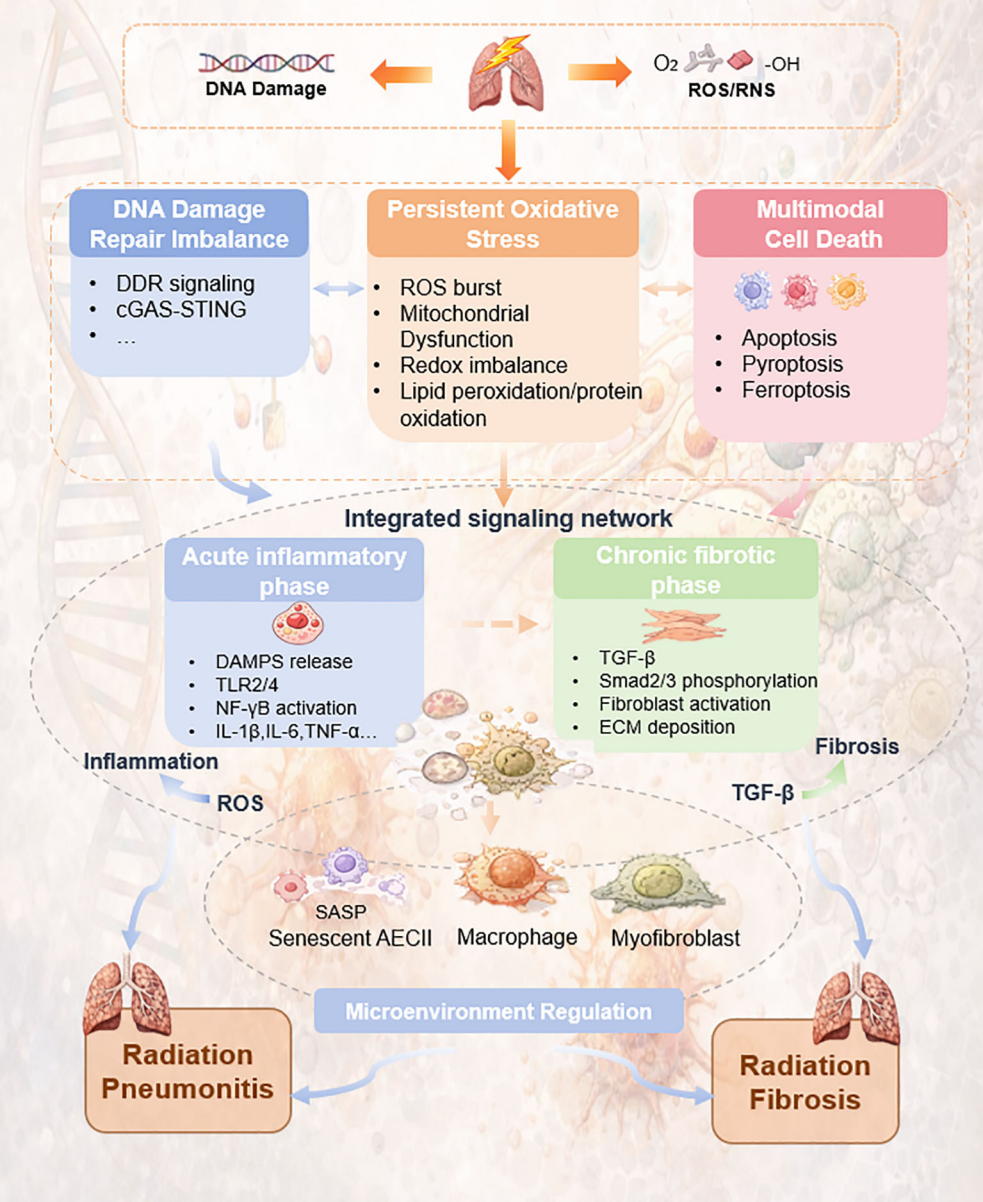
Schematic diagram illustrating the molecular mechanisms and network interactions of radiation-induced lung injury.

In summary, RILI is a prevalent and serious adverse effect of thoracic radiotherapy, whose pathogenesis is driven by a web of interconnected mechanisms. These include flawed DNA repair, oxidative stress, intertwined inflammatory and fibrotic cascades, cellular senescence, and immune dysregulation. Rather than a linear pathway, RILI manifests as a complex, self-reinforcing pathological network. The molecular mechanisms and network interactions underlying RILI are summarized in **Figure 1**. Deciphering the intricate connections within this network is paramount for identifying novel therapeutic targets and developing effective strategies to prevent and treat this condition. Translating these mechanistic insights into clinical practice presents several challenges. Firstly, therapeutic interventions must account for temporal and spatial dynamics, as the dominant pathological pathways shift over the course of RILI. This necessitates precise timing of interventions, such as deploying anti-inflammatory agents in the acute phase and anti-fibrotic later. Secondly, the relative contribution of different cell populations—such as alveolar epithelial cells, fibroblasts, and immune subsets to RILI progression remains debated, particularly in the context of emerging single-cell and spatial omics data, which sometimes yield inconsistent interpretations across models and species. Thirdly, emerging regulatory layers beyond classical cytokine and stress-response pathways deserve greater attention in future RILI research. These include epitranscriptomic mechanisms, such as m6A RNA modification, which may influence epithelial plasticity, fibroblast activation, and inflammatory signaling, as well as biomolecular phase separation, which may reorganize stress-responsive signaling complexes and transcriptional programs after irradiation. Lastly, the development of personalized risk assessment and prevention strategies, based on biomarkers predicting individual risk, will enable individualized radiotherapy planning. By integrating these multifaceted strategies, the future management of RILI can evolve from supportive care to proactive, mechanism-driven precision medicine, which could provide crucial support for enhancing treatment outcomes and quality of life in thoracic cancer patients.
